# Early Conversion to Once-Daily MeltDose^®^ Extended-Release Tacrolimus (LCPT) in Liver Transplant Patients

**DOI:** 10.3390/jcm14238530

**Published:** 2025-12-01

**Authors:** Leonie S. Jochheim, Anne Hörster, Alexandra Frey, Kerstin Herzer, Dieter Paul Hoyer, Knut M. Nowak, Ulf P. Neumann, Hartmut Schmidt, Jassin Rashidi-Alavijeh, Moritz Passenberg, Katharina Willuweit

**Affiliations:** 1 Department of Gastroenterology, Hepatology and Transplant Medicine, University Hospital Essen, University of Duisburg-Essen, 45147 Essen, Germany; leonie.jochheim@uk-essen.de (L.S.J.);; 2 Department of Nuclear Medicine, University Hospital Essen, University of Duisburg-Essen, 45147 Essen, Germany; 3 Knappschafts-Klinik Bad Neuenahr, 53474 Bad Neuenahr-Ahrweiler, Germany; 4 Department of General, Visceral, Vascular and Transplant Surgery, University Hospital Essen, University of Duisburg-Essen, 45147 Essen, Germany

**Keywords:** liver transplant, early conversion, tacrolimus, MeltDose^®^ extended-release tacrolimus (LCPT), adherence, renal function

## Abstract

**Background:** Switching stable liver transplant (LT) recipients from twice-daily immediate-release tacrolimus (IR-Tac) to once-daily MeltDose^®^ extended-release tacrolimus (LCPT) has been proven safe and well tolerated. Moreover, the switch has been associated with enhanced treatment adherence, improvement of tremors, and preserved renal function. Here, we hypothesized that switching to LCPT early after LT may enhance long-term patient outcomes significantly. **Methods:** This single-center, observational study investigated the long-term safety of LCPT in a large cohort of LT recipients (*n* = 100). Allograft function, emerging adverse events, the incidence of rejection reactions, renal function, lipid and glucose metabolism, and treatment adherence were assessed over 24 months. **Results:** In 56% of patients, the switch was conducted within 4 weeks post-transplantation. Adverse events occurred in 90% of patients during the 24-month follow-up, including gastrointestinal complications (28%), neurological symptoms (28%), skin disorders (26%), metabolic disorders (22%), and fatigue (18%). Seven patients (7%) developed renal insufficiency, and five patients (5%) developed renal failure. Three episodes of chronic graft rejection reactions (3%) and a single transplant failure (1%) were observed over 24 months. LCPT was discontinued in 10 patients. Liver and renal function markers, blood lipids (cholesterol and triglycerides), and glucose levels remained stable over the 24-month follow-up. However, 58% of LT recipients had one of their liver function markers elevated at baseline (i.e., before the switch), 28% had low glomerular filtration rate (GFR < 60 mL/min/1.73 m^2^), and 18% had high serum creatinine (>1.3 mg/dL). In these subgroups, the early switch to LCPT was associated with a significant decrease in liver enzymes (*p* < 0.001 for alanine transaminase; *p* = 0.032 for gamma-glutamyl transferase; and *p* < 0.001 for total bilirubin) and a significant decrease in serum creatinine levels (*p* < 0.001). Self-reported treatment adherence was good and consistent throughout the study. **Conclusions:** The early switch from IR-Tac to LCPT was safe and effective in our cohort and may be particularly beneficial for patients with suboptimal liver and renal function following LT.

## 1. Introduction

Allograft rejection remains an important cause of morbidity and mortality in liver transplant (LT) recipients [1]. Calcineurin inhibitors (CNIs) reduce the risk of T-cell-mediated rejection and are an essential component of immunosuppression therapy [2]. Among CNIs, tacrolimus has been proven more effective than cyclosporine in preventing acute rejections and mortality, and is therefore the first-line CNI for maintenance immunosuppression therapy after LT [3,4,5].

Tacrolimus has a narrow therapeutic range and shows high variability in pharmacokinetics [6]. Subtherapeutic levels increase the risk of graft rejection, while supratherapeutic concentrations are associated with adverse reactions, including cardiovascular adverse events, nephrotoxicity, neurotoxicity, and hyperlipidemia [2,4]. Systemic tacrolimus levels are influenced by several factors, such as polymorphisms in the genes of the cytochrome P450 (CYP) 3A subfamily, concomitant medications, non-adherence to immunosuppression treatment regimens, and the type of tacrolimus formulation [7,8,9,10]. Close monitoring of blood tacrolimus levels is essential to maintain therapeutic efficacy while minimizing toxicity [11].

Three different types of tacrolimus formulations have been developed [6,10]. Immediate-release tacrolimus (IR-Tac, Astellas Pharma, Tokyo, Japan) was the first approved formulation and is characterized by a low bioavailability requiring twice-daily administration, fluctuations in systemic concentrations, and transient supratherapeutic levels around the maximum concentration (C_max_) peak [10,12]. A once-daily, prolonged-release tacrolimus (PR-Tac, Astellas Pharma GmbH, Munich, Germany) formulation was developed subsequently [13]. PR-Tac simplifies treatment and improves adherence [14]. However, it still displays pharmacokinetic fluctuations and does not prevent the supratherapeutic tacrolimus levels around the C_max_ peak [6,10]. Indeed, patients switching from IR-Tac to PR-Tac usually require higher tacrolimus doses to achieve the target trough levels, especially in the early post-transplant period [15]. Higher dose requirements further raise peak levels [10], increasing the risk of peak-related toxicities, such as adverse neurologic events (e.g., tremors) and, potentially, impaired renal function [10,16,17,18,19].

LCP tacrolimus (LCPT, Envarsus XR, Chiesi Farmaceutici, Parma, Italy) is the most recent tacrolimus formulation approved in LT [20]. It is formulated using MeltDose^®^ technology, a process that reduces bulk tacrolimus to the smallest possible particle size (<100 nm) to be further organized as a solid suspension into oral tablets [21]. This technology enables the sustained release of submicron tacrolimus particles over the length of the gastrointestinal tract. LCPT displays higher bioavailability than IR-Tac and PR-Tac, as evidenced by LCPT’s ability to achieve equivalent target trough levels with 30–38% dose reduction [10,18,22,23,24]. Moreover, pharmacokinetic studies have shown that LCPT displays less peak-to-trough fluctuations and avoids supratherapeutic tacrolimus concentrations around the maximum concentration peak [6,10,25,26].

Previous studies have demonstrated the feasibility of converting stable LT recipients from IR-Tac and PR-Tac to LCPT [17,18,23,26,27]. Along with good tolerability and dose reductions, preserved renal function, significant improvement of tremors, and better treatment adherence have been reported in stable LT recipients [17,18,27]. Based on these observations, we hypothesized that LT recipients would benefit from switching to LCPT early after transplant. Nevertheless, the safety and tolerability of switching early to LCPT have not yet been fully established. We therefore investigated the tolerability and effectiveness of the early switch (i.e., within a few weeks after transplant) from twice-daily IR-Tac to LCPT in a large cohort of LT recipients. Allograft function, emerging adverse events, and the incidence of rejection reactions were recorded over 24 months. Tacrolimus dose and trough levels, renal function, lipid and glucose metabolism, and treatment adherence were also assessed.

## 2. Materials and Methods

This was a single-center, prospective, observational study enrolling 100 consecutive patients from the Outpatient Clinic of the University Hospital Essen, Germany, who were converted to LCPT (Envarsus^®^, Chiesi Farmaceutici S.p.A., Parma, Italy) after LT. Patients were converted for the purpose of prophylactic neuroprotection and nephroprotection. All patients converted to LCPT during the study period were enrolled consecutively, while the timing of conversion was individually determined by the treating physicians as part of routine clinical care.

The study enrolled patients from March 2017 through March 2020 and was conducted in compliance with the Ethics Committee of the University Hospital Essen [File number 16-6754-BO], the Declaration of Helsinki (6th revision 2008), and the International Conference on Harmonization (ICH) Good Clinical Practice (GCP) Guidelines.

Adult recipients (≥18 years old) of LT receiving non-LCPT CNI immunosuppression (i.e., IR-Tac or PR-Tac) were eligible for conversion to LCPT if they provided informed consent to participate in the study. Patients under 18 years of age were excluded. Only stable patients without documented prior acute rejection episodes were included, as the early conversion strategy was specifically designed for patients with uncomplicated post-transplant courses.

The primary aim of the study was to assess tolerability and graft function over 24 months after switching to LCPT within a few weeks after liver transplantation. Other outcomes included renal function, glucose and lipid metabolism, and treatment adherence.

Patient care and data collection were performed according to the routine program of the Outpatient Clinic of the University Hospital Essen. Target trough tacrolimus levels in our center are between 5 and 10 ng/mL in the first year and 4–6 ng/mL thereafter, depending on combinations with other immunosuppressants. All patients initially received standard immunosuppressive therapy consisting of a calcineurin inhibitor (IR-tacrolimus or cyclosporine), mycophenolate mofetil (MMF), and corticosteroids, without the use of induction agents. According to the product characteristics, the conversion ratio from IR-Tac to LCPT is 1:0.7 (mg:mg). In patients converting from cyclosporine, the recommended initial dose of LCPT is 0.17 mg/kg/day.

Age, gender, transplantation date, and the indication for transplantation were documented at baseline. In addition, adverse events, level of immunosuppression, kidney (serum creatinine and glomerular filtration rate [GFR]) and liver function (aspartate transferase [AST], alanine transaminase [ALT]), gamma-glutamyl transferase [GGT], alkaline phosphatases [AP], and total bilirubin), lipid status (cholesterol, triglycerides), and blood sugar levels were documented at baseline, and subsequently, at 4 weeks, 8 weeks, 12 weeks, 6 months, 12 months, and 24 months after switching to LCPT. Adverse events were recorded cumulatively throughout the study period, though the exact onset timing of individual adverse events was not uniformly documented. Episodes of graft rejection reactions were confirmed by biopsy.

Changes in medical adherence were assessed using the Essen Compliance Score (ECS) questionnaire, administered at four weeks and one year after switching to LCPT. The ECS tool is a modified version of the Morisky score [28,29] and was validated for patients after kidney transplantation in our university hospital. A score of 0 indicates perfect compliance, and a score > 7 indicates low adherence.

No formal size calculation was performed. A sample size of 100 patients was deemed appropriate based on the feasibility of our center and the previous experience from similar studies. Tabular data are either presented as the number and percentage or as the median and the 95% confidence interval (CI) or the range, as appropriate. The boxplots display the median and the 95% CI of each parameter. Standard values or limit ranges for the various laboratory parameters are indicated in the boxplots by dashed lines. Outliers were not included in the boxplots, although they were included in the statistical analyses. Multiple grouped variables were analyzed using ANOVA repeated measurements. A *p* < 0.05 was accepted as statistically significant.

## 3. Results

### 3.1. Patient Disposition and Baseline Characteristics

**Figure 1:**
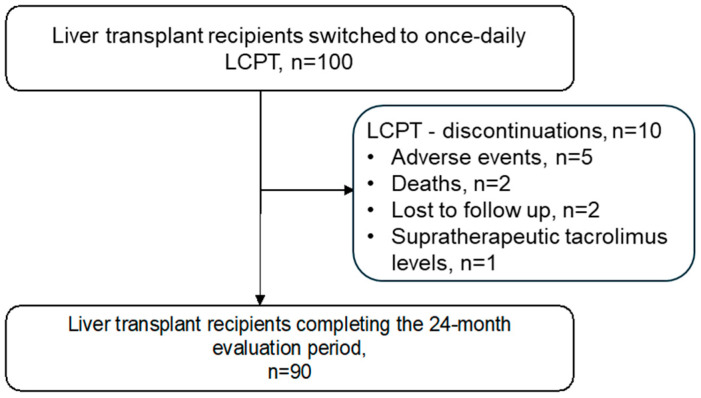
Patient disposition.

The cohort comprised a total of 100 patients (Figure 1), and 90 completed the 24-month follow-up. Sixty-three percent of patients were male, the median age was 52 years, and the median body mass index (BMI) was 23 kg/m^2^ (Table 1). One patient (1%) received cyclosporine-based immunosuppression prior to switching to LCPT. The rest (99%) were switched from IR-Tac to LCPT at a median of 27 days after transplantation. Of these, 56 patients were switched within 30 days (median 21 days; range: 10–30 days), 31 within 31 and 100 days (median 60 days; range: 32–100 days), and 13 between 101 and 276 days (median 136 days; range: 101–276 days).

Autoimmune liver diseases (27%) and alcoholic cirrhosis (Alcoholic Steatohepatitis, 21%) were the most common indications for LT. Other causes were acute liver failure (8%), hepatocellular carcinoma (HCC) in 17 patients (17%: 8 patients with HCC alone, 8 with HCC due to hepatitis C virus, and 1 with HCC due to hepatitis B virus), and non-alcoholic steatohepatitis (4%).

### 3.2. Effectiveness, Adverse Events and Causes of LCPT Discontinuation

Three episodes of rejection reactions (3%) and one transplant failure (1%) were observed over the entire observation period of 24 months. One of the rejection reactions was attributed to the patient’s non-adherence to immunosuppression, as reported by the patient (Table 2). At 24 months, adverse events had occurred in 90% of patients. Gastrointestinal complications such as diarrhea, constipation, or abdominal pain and neurological symptoms such as headache, migraine, sleep disorders, tremor, or dizziness were observed in 28% of patients. Skin and subcutaneous tissue disorders, including alopecia, pruritus, or dry skin, occurred in 26% of patients. Adverse events were recorded cumulatively throughout the 24-month observation period. The majority of adverse events occurred during the first 3 months post-conversion. Notably, 7 patients (7%) developed renal insufficiency (defined as eGFR decline of >20% from baseline but >30 mL/min/1.73 m^2^) and 5 patients (5%) developed renal failure (defined as eGFR < 30 mL/min/1.73 m^2^) during follow-up. Among patients with renal insufficiency, the median eGFR at study endpoint was 52 mL/min/1.73 m^2^ with median serum creatinine of 1.48 mg/dL. Of the 5 patients with renal failure, 2 required intermittent dialysis during the study period. LCPT dosages were adjusted in response to adverse events, particularly neurological symptoms, with more frequent dose modifications observed during the first 3 months post-conversion.

Immunosuppression with LCPT was discontinued in ten patients (10%). Two patients died: one due to acute liver failure at 12 months and another due to multi-organ failure in the context of pneumogenic sepsis at 6 months. Five patients discontinued treatment due to side effects (hair loss, pruritus, tremor; alopecia areata, reduced finger sensitivity, restlessness and sleep disorders). One patient discontinued due to persistent high levels of tacrolimus. Two patients were lost to follow-up due to a site change.

### 3.3. Liver Graft Function

**Figure 2:**
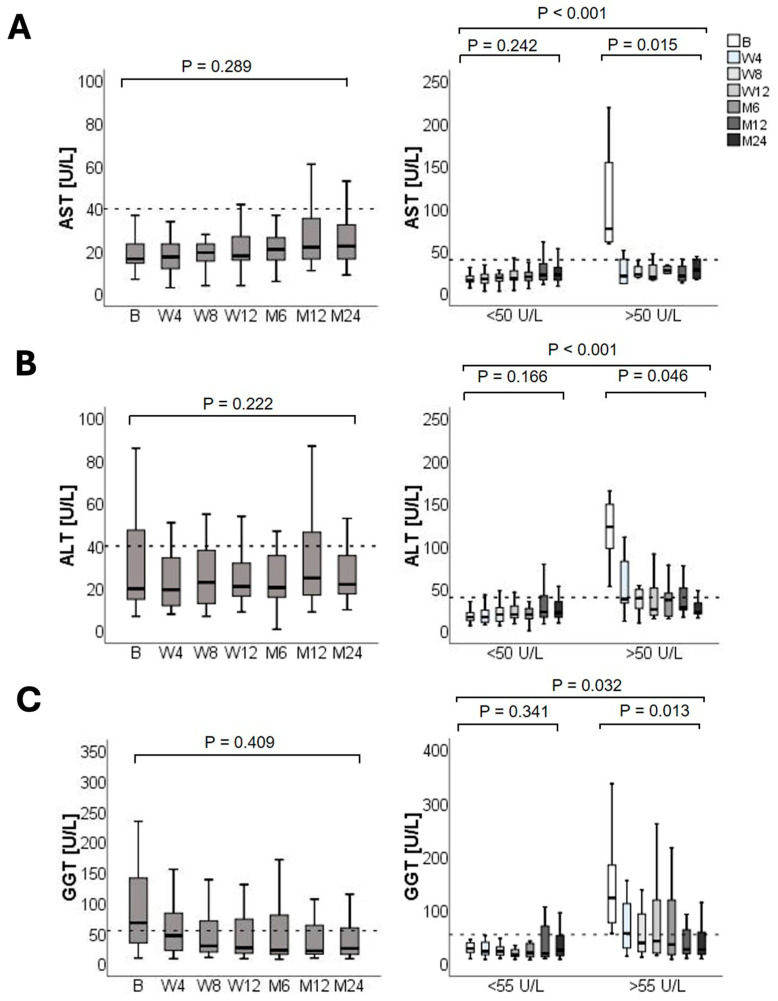
Liver transaminases over the 24-month follow-up after switching to LCPT. (**A**) Aspartate aminotransferase, (AST), (**B**) alanine aminotransferase (ALT), and (**C**) gamma-glutamyl transferase (GGT) levels in the overall population (**left**), and according to normal or elevated values at baseline (**right**). Boxplots show median values with 95% confidence interval. B, baseline; M, month; W, week. The dashed horizontal lines indicate the established upper limit of normal (ULN) for the respective laboratory parameters, serving as reference values for clinical interpretation.

Liver transaminases remained stable throughout the study period (Figure 2). AST levels showed a non-significant slight increase over the 24-month follow-up, whereas ALT remained stable, and GGT showed a non-significant downward trend. Six patients had elevated AST levels (i.e., >50 U/L) at baseline. Conversely, 22 and 58 patients had elevated ALT and GGT levels at the time of switching from IR-Tac to LCPT. The early switch to LCPT significantly improved AST, ALT, and GGT levels. In these patients, the decrease in liver enzyme levels was already observed during the first visit following the switch to LCPT (i.e., at week 4) and persisted for the 24-month follow-up.

**Figure 3:**
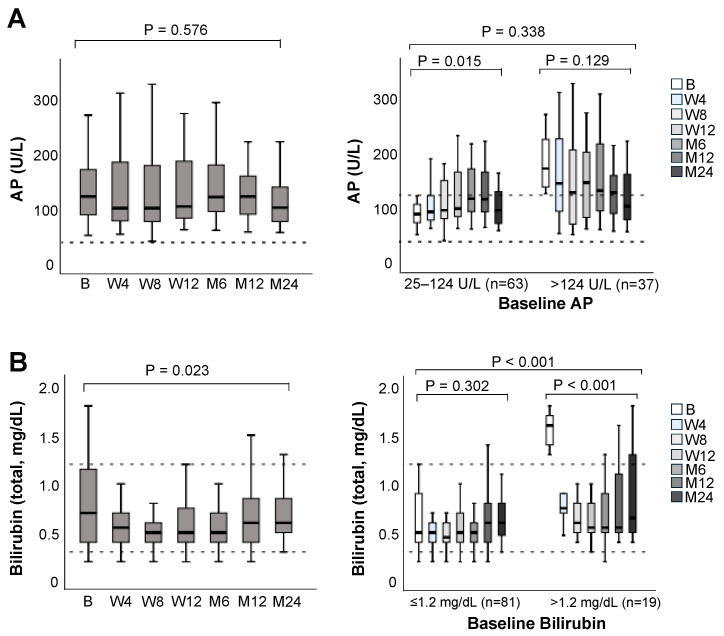
(**A**) Alkaline phosphatase (AP) and (**B**) bilirubin levels over the 24-month follow-up after switching to LCPT in the overall population (**left**), and according to normal or elevated values at baseline (**right**). Boxplots show median values with 95% confidence interval. B, baseline; M, month; W, week. The dashed horizontal lines represent the established reference range (lower and upper limits of normal) for the respective laboratory parameters, providing guidance for clinical relevance.

Regarding cholestasis, no change was observed for AP over 24 months in the overall population (*p* = 0.576) (Figure 3A). Sixty-three patients had normal AP levels at baseline. After switching to LCPT, this subgroup experienced an upward trend in the median AP values over the first year, which, however, returned to baseline at month 24 (*p* = 0.015). Thirty-seven patients had elevated AP values at baseline. This subgroup showed a non-significant downward trend over the 24-month follow-up, with the median AP returning to reference values by month 24.

Median bilirubin remained within range for the whole observational period (Figure 3B). Median values decreased over the first six months and slightly increased thereafter (*p* = 0.023). A subgroup of 19 patients had bilirubin values > 1.2 mg/dL at the time of switching to LCPT. The median bilirubin of this subgroup already dropped to reference values by the first visit (at week 4) and remained within range for the entire follow-up period (median [range] baseline bilirubin: 1.6 mg/dL [1.3–2.5] Vs. At 24 months: 0.75 mg/dL range [0.4–1.8], *p* < 0.001).

### 3.4. Renal Function

**Figure 4:**
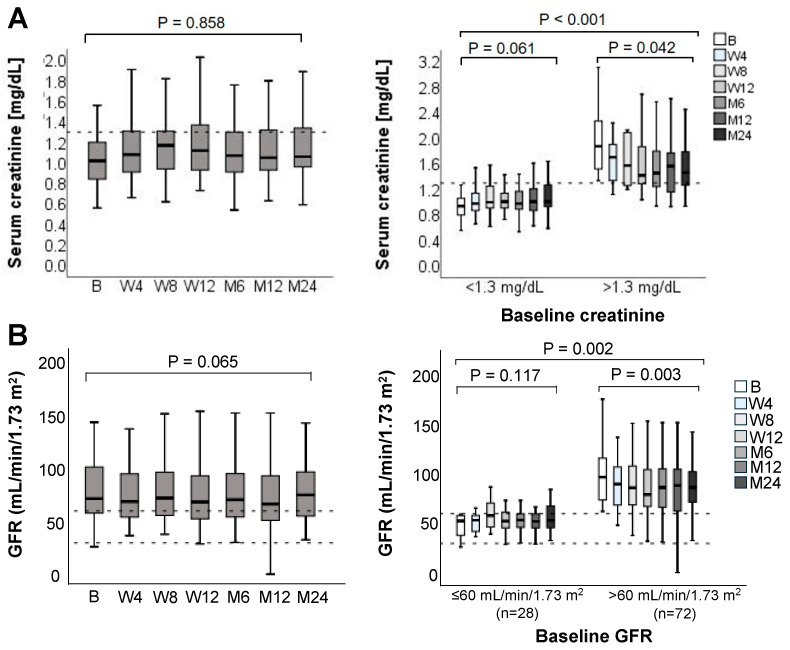
(**A**) Serum creatinine levels and (**B**) glomerular filtration rate over the 24-month follow-up after switching to LCPT in the overall population (**left**), and according to normal or elevated values at baseline (**right**). Boxplots show median values with 95% confidence interval. B, baseline; M, month; W, week. The dashed horizontal lines represent the established reference range (lower and upper limits of normal) for the respective laboratory parameters, providing guidance for clinical relevance.

Throughout the 24-month observation period, renal function remained stable in the overall population, as indicated by unchanged median serum creatinine concentrations and glomerular filtration rates (Figure 4). In the subset of patients (*n* = 18) with elevated baseline serum creatinine levels, a significant reduction was observed over 24 months (median [range] serum creatinine at baseline: 1.87 mg/dL [1.39–3.11] Vs. At 24 months: 1.51 mg/dL [0.94–2.59], *p* = 0.042). Median GFR values remained stable over the study period in patients with impaired renal function at baseline (*n* = 28; i.e., GFR < 60 mL/min/1.73 m^2^ at the time of conversion to LCPT). Patient subgroups with normal serum creatinine and GFR values at baseline experienced a slight decline in renal function the 24-month follow-up period.

### 3.5. Lipid and Glucose Metabolism

**Figure 5:**
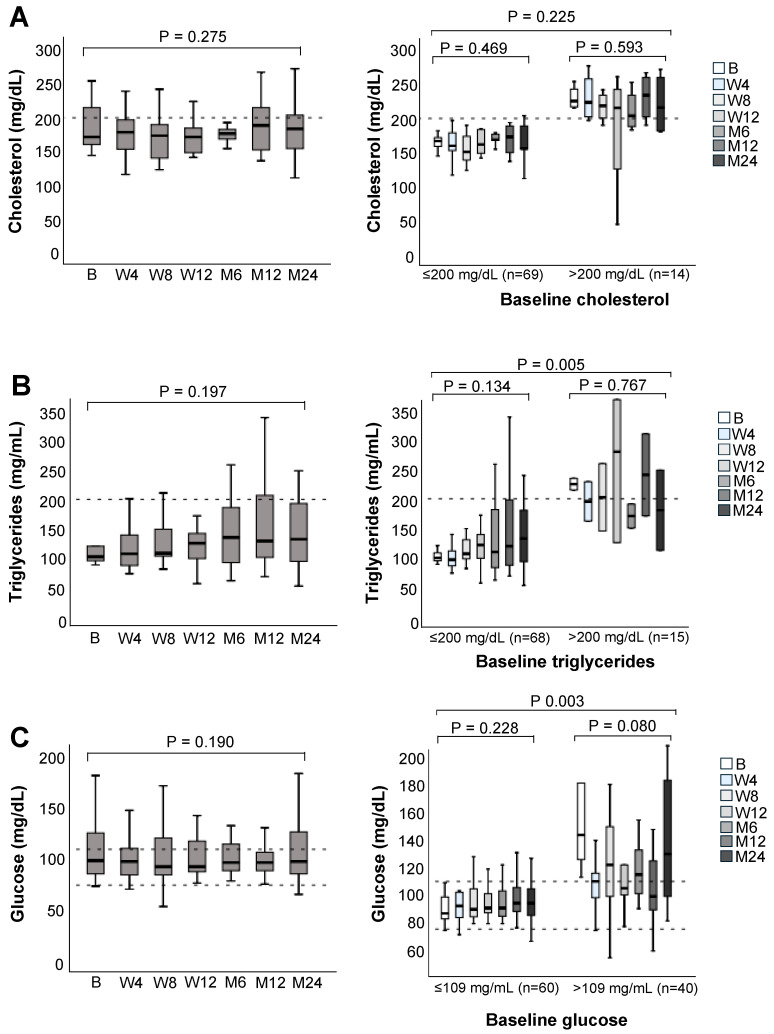
(**A**) Cholesterol, (**B**) trygliceride, and (**C**) glucose levels over the 24-month follow-up after switching to LCPT in the overall population (**left**), and according to normal or elevated values at baseline (**right**). Boxplots show median values with 95% confidence interval. B, baseline; M, month; W, week. The dashed horizontal lines represent the established reference range (lower and upper limits of normal) for the respective laboratory parameters, providing guidance for clinical relevance.

In the overall population, median cholesterol, triglyceride, and glucose levels remained stable and within their ranges over the course of the study (Figure 5). Eighteen, 15, and 40 patients had elevated cholesterol, triglyceride and glucose values at baseline, respectively. Median cholesterol values remained elevated over the 24-month follow-up in the subgroup of patients with high cholesterol (>200 mg/dL) at baseline. Median triglyceride and glucose values decreased initially in patients with high baseline values, although both fluctuated over the observational period.

### 3.6. Tacrolimus Trough Levels

**Figure 6:**
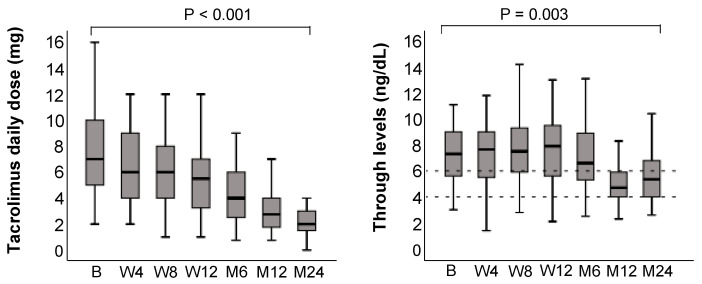
Tacrolimus daily dose (**left**) and trough levels (**right**) over the 24-month follow-up after switching to LCPT. Boxplots show median values with 95% confidence interval. B, baseline; M, month; W, week. The dashed horizontal lines represent the established reference range (lower and upper limits of normal) for the respective laboratory parameters, providing guidance for clinical relevance.

The total tacrolimus dose was significantly reduced over the course of the observational period, from 8 mg [2–24] at baseline to 2.0 mg [0.75–10.75] at 24 months (*p* < 0.001, Figure 6). Median tacrolimus trough levels decreased from 7.8 ng/dL [2.2–43.9] at baseline to 5.3 ng/dL [1.5–11.2] at 24 months (*p* = 0.003).

### 3.7. Adherence to Treatment

The ECS is a validated instrument for assessing adherence in transplant recipients, originally developed as an extended version of the Morisky Score to address its limitations, including categorical responses and insufficient item depth. The ECS includes 24 items rated on a Likert scale, of which 18 contribute to the total score (range: 0–72), with lower scores indicating better adherence.

In our cohort, four weeks after conversion to LCPT, adherence was categorized as ‘good’ based on the ECS, with a median score of 3 (range: 0–13). At 24 months, the median score was sustained (range: 0–18, Figure 7), falling within the ‘excellent adherence’ category. According to ECS classification, scores between 0 and 3 reflect excellent adherence, and scores between 3 and 7 indicate good adherence. These findings suggest sustained and potentially improved adherence over time following the switch to LCPT.

## 4. Discussion

In this observational, single-center study, we analyzed the outcomes of converting liver transplant recipients from IR-Tac to once-daily LCPT. Most patients underwent conversion within the first four weeks post-transplantation. LCPT was well tolerated, with only three biopsy-proven acute rejection episodes and a single instance of graft loss documented over the 24-month follow-up period. Notably, one rejection episode was associated with documented non-adherence to immunosuppressive therapy. No new safety signals emerged throughout the study duration, and treatment discontinuation due to adverse events occurred in only 5% of patients. Overall, the incidence of adverse events, treatment discontinuation, and mortality following conversion to LCPT was consistent with previously published data on tacrolimus-based immunosuppression in liver transplantation [14,24,27,30,31,32].

In addition to its favorable safety profile, patients who converted to LCPT showed a significant improvement in transaminase and bilirubin levels among those who presented with elevated liver enzymes at baseline. Soliman et al. have also recently reported reductions in liver enzyme levels in LT recipients initiated de novo with LCPT. In their study, mean AST, ALT, and GGT appeared elevated at day 7 (204, 130, and 48 U/L, respectively) but their mean values significantly decreased over the first 90 days post-LT [32]. In the present cohort, median AST and ALT levels were within normal limits at baseline, whereas median GGT values were mildly elevated. However, a substantial proportion of patients—22% and 58%, respectively—presented with elevated ALT and GGT levels at the time of conversion to LCPT, suggestive of subclinical graft dysfunction or biliary complications. In our prior study conducted on stable LT recipients, only 10.7% and 19.8% of patients exhibited elevated ALT and GGT values at baseline, respectively. Following conversion from IR-Tac to LCPT, median transaminase levels demonstrated a continuous decline over the 24-month follow-up period [23]. The significant reduction in liver enzyme levels observed in the present study may, in part, be attributable to the pharmacokinetic properties of LCPT. However, this improvement may also reflect the physiological normalization of graft function following the transient elevation in liver enzymes commonly observed during the early postoperative period after liver transplantation [33]. Furthermore, given that patients in our cohort were converted to LCPT at different postoperative time points—ranging from early (≤30 days) to intermediate (31–100 days) and late (>100 days)—the heterogeneous timing of conversion introduces variability in the postoperative course, further limiting causal inference. Therefore, it can be concluded that the potential contribution of LCPT should be regarded as hypothesis-generating, as opposed to being demonstrable, in accordance with the study’s design.

The renal outcomes observed in this study warrant careful consideration. While overall renal function parameters remained stable in the cohort, 7 patients (7%) developed renal insufficiency and 5 patients (5%) experienced renal failure during the 24-month follow-up period. Among patients with renal insufficiency (*n* = 7), the median eGFR at study endpoint was 52 mL/min/1.73 m^2^ with median serum creatinine of 1.48 mg/dL. Of the 5 patients with renal failure, 2 required intermittent dialysis support, though none underwent kidney transplantation.

Long-term use of CNIs as maintenance immunosuppression in LT recipients is associated with progressive renal impairment, cardiovascular complications, and an increased incidence of malignancies [2]. Nephrotoxic effects have been reported in up to 70% of LT patients, and approximately 18% develop end-stage renal disease within five years post-transplantation [34,35,36]. High tacrolimus metabolizers require higher daily doses to achieve target trough levels. This leads to disproportionately elevated peak concentrations, which have been implicated in increased nephrotoxicity and other tacrolimus-related toxicities [18,30].

LCPT can potentially reduce nephrotoxicity by providing lower tacrolimus peak levels and achieving target trough levels at lower doses. Von Einsiedel et al. reported a 50% improvement in the concentration-to-dose (C/D) ratio over 12 months in stable LT recipients converted from PR-Tac to LCPT compared to patients maintained on PR-Tac [18]. Furthermore, the LCPT cohort demonstrated significantly higher glomerular filtration rates (GFR) at 3, 6, and 12 months post-conversion [18]. The MOTTO trial randomized 105 liver transplant recipients to receive either de novo LCPT or PR-Tac and evaluated the incidence of a composite primary endpoint—post-transplant diabetes mellitus, hypertension, and chronic kidney disease (defined as GFR < 60 mL/min/1.73 m^2^)—at 12 months post-transplantation [31]. In the intention-to-treat analysis, the incidence of the composite outcome was significantly lower in the LCPT group. According to the authors, LCPT exerted a favorable effect on renal function, with a 15.9% to 18.2% absolute reduction in the prevalence of chronic kidney disease persisting for more than three months after transplantation [31].

In contrast to the favorable renal outcomes reported in the MOTTO trial, Soliman et al. observed a slight, albeit non-significant, increase in serum creatinine levels over a 6-month period in LT recipients treated de novo with LCPT [32]. In our cohort, overall renal function remained stable throughout the 24-month follow-up. However, a distinct subgroup of patients presented with impaired renal function at baseline—defined by serum creatinine > 1.3 mg/dL and GFR < 60 mL/min/1.73 m^2^—while still receiving IR-Tac. In these patients, conversion to LCPT was associated with a significant reduction in serum creatinine and stable GFR over the study period, suggesting a potential nephroprotective effect of LCPT in the setting of pre-existing renal dysfunction. Conversely, patients with normal renal function at baseline experienced a modest increase in serum creatinine and a decline in GFR over 24 months, likely reflecting the expected trajectory of renal function deterioration commonly observed in LT recipients over time. The higher incidence of renal complications in our early-conversion cohort compared to late-conversion studies may reflect several factors: first, the early post-transplant period is associated with inherent renal vulnerability due to perioperative factors; second, the early conversion timing may not provide the same degree of protective benefits observed in stable, late-conversion cohorts. Further investigation is needed to determine optimal timing for LCPT conversion to maximize nephroprotective benefits while minimizing risks. The fact that LCPT dosages were adjusted in response to adverse events, particularly during the first 3 months post-conversion, suggests that closer monitoring and more conservative initial dosing may be warranted in early post-transplant conversions.

Neurological symptoms, observed in 28% of patients, may have influenced LCPT dosage adjustments particularly during the first 3 months post-conversion. While the exact temporal distribution of adverse events was not uniformly recorded, clinical practice at our center typically involves closer monitoring and more conservative dose adjustments in the early post-conversion period when such symptoms manifest. This pragmatic approach to dose management may have contributed to the overall tolerability profile observed in our cohort.

Long-term exposure to immunosuppressive therapy is also associated with the development of hyperlipidemia and altered glucose metabolism [37]. Post-liver transplantation diabetes mellitus (PLTDM) develops in up to 30% of LT recipients and is associated with an increased risk of mortality and multiple morbid outcomes [38]. In the present study, median glucose levels remained stable over 24 months in the overall cohort. Notably, 40% of the patients showed impaired glucose metabolism (blood glucose > 109 mg/dL) at baseline. This subgroup displayed a median baseline glucose value of 140 mg/dL, above the 126 mg/dL threshold to diagnose PLTDM [38]. Median glucose levels decreased in this subgroup after switching to LCPT, falling below the PLTDM threshold in the first year but reaching borderline values at the end of the 24-month follow-up. Median cholesterol and triglyceride levels remained fairly stable over the study period. Patients with high cholesterol (>200 mg/dL) at baseline showed persistently high levels also at 24 months, while patients with elevated triglycerides at baseline displayed fluctuations above and below the 200 mg/dL threshold at different visits.

LT recipients face the lifelong challenge of managing complex immunosuppressive regimens, often associated with a high pill burden and substantial adverse effects. Non-adherence remains a common and clinically relevant issue in this population, representing a key modifiable risk factor for both acute and chronic graft rejection, graft loss, and mortality [27]. In the present study, self-reported adherence was consistently high throughout the observation period. Notably, median ESC values improved from the ‘good adherence’ range four weeks after conversion to LCPT to the ‘excellent adherence’ range at one year.

The present study is subject to several limitations, which must be considered when interpreting the findings. Most notably, the absence of a control group limits the ability to draw definitive conclusions about the superiority of LCPT over IR-Tac. Whilst the incorporation of consecutive patients helps to minimize selection bias, several sources of selection bias remain. First, the timing of conversion to LCPT was not standardized but was individually determined by treating physicians based on clinical judgment (median 27 days post-transplant, range 10–276 days). This variability in conversion timing represents a significant limitation, as patients converted at different time points may have had different risk profiles and clinical trajectories. Second, patients were selected for conversion primarily for prophylactic neuroprotection and nephroprotection purposes, which may have introduced selection bias toward patients perceived to be at higher risk for these complications. Third, only patients without prior acute rejection episodes were included in this study, which represents an important selection bias. The exclusion of patients with prior rejection may limit the generalizability of findings to the broader post-transplant population and may have contributed to the favorable safety profile observed. The applicability of these results to patients with a history of acute rejection remains unclear and warrants further investigation. The single-center setting further limits the external validity and generic applicability of the findings. A non-converted comparator cohort was not included in the study because only a very small and highly heterogeneous minority of patients remained on IR-Tac, primarily due to individual clinical reasons that would have introduced substantial confounding by indication. Furthermore, the timing and documentation of adverse events present additional limitations. Table 2 reports cumulative adverse events over the 24-month period without specifying the temporal distribution of these events, making it difficult to assess whether adverse events were concentrated in particular time periods or whether LCPT dosage adjustments were made in response to specific adverse events. Additionally, ECS was not administered prior to conversion to LCPT, and therefore longitudinal changes in adherence behavior before and after the switch could not be assessed.

## 5. Conclusions

Early conversion from IR-Tac to LCPT was well tolerated in our cohort, with only a few episodes of graft rejection and a single case of graft loss reported during the 24-month follow-up period. Overall, liver and renal function, as well as glucose and lipid metabolism, remained stable across the study population. Notably, in patients with impaired hepatic or renal function at the time of conversion, early LCPT initiation was associated with significant improvements in liver enzyme profiles and serum creatinine levels. Adherence to LCPT was high following the switch and remained consistent throughout the follow-up.

## Figures and Tables

**Figure 7:**
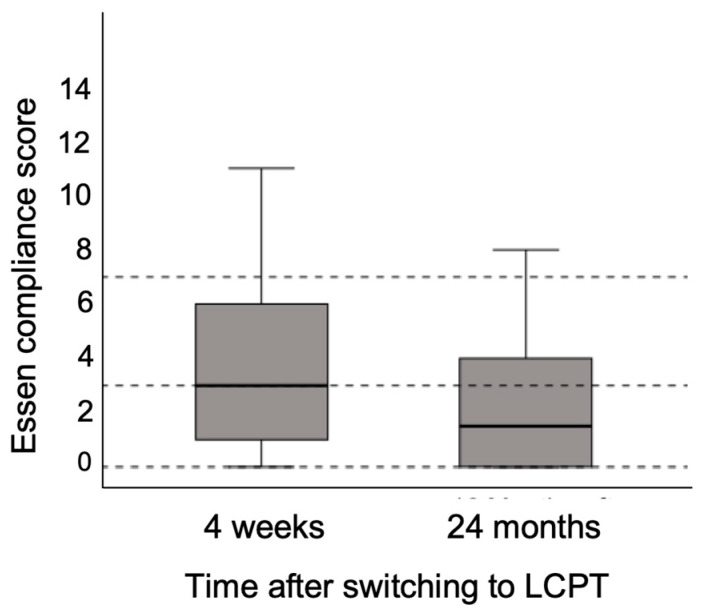
Development of the ECS within 24-month follow-up after switching to LCPT. Boxplots show median values with 95% confidence interval.

**Table 1:** Baseline characteristics and indications for liver transplantation.

Baseline Characteristics	LCPT (*n* = 100)
Recipient demographics	
Gender, male, *n* (%)	63 (63)
Age, years, median [range]	52 [18–69]
Switch to LCPT, days, median (range)	27 [10–276]
BMI, kg/m^2^, median (range)	23 [16–36]
Indication for liver transplantation, *n* (%)	
Autoimmune liver diseases (PBC, PSC, AIH)	27 (27)
Alcoholic steatohepatitis	21 (21)
Acute Liver Failure	8 (8)
Hepatocellular carcinoma (HCC)	8 (8)
HCC due to hepatitis C Virus	8 (8)
HCC due to hepatitis B virus (HBV)	1 (1)
Non-alcoholic Steatohepatitis	4 (4)
Cryptogenic cirrhosis of the liver	4 (4)
HBV	4 (4)
Autosomal dominant polycystic liver disease	4 (4)
Secondary sclerosing cholangitis	3 (3)
Budd-Chiari syndrome	2 (2)
HCV and HBV	1 (1)
Other	5 (5)

AIH, Autoimmune Hepatitis; BMI, Body Mass Index; LCPT, Once-daily MeltDose^®^ Extended-Release Tacrolimus; PBC: Primary Biliary Cholangitis, PSC: Primary Sclerosing Cholangitis.

**Table 2:** Adverse events and causes for LCPT discontinuation registered in the study.

Adverse Events, *n* (%)	LCPT (*n* = 100)
Rejection reaction	3 (3)
Transplant failure	1 (1)
Renal insufficiency	7 (7)
Renal failure	5 (5)
Gastrointestinal complications	28 (28)
Neurological symptoms	28 (28)
Skin and subcutaneous tissue disorders	26 (26)
Metabolism and nutrition disorders	22 (22)
Fatigue	18 (18)
Procedural complications	17 (17)
Blood count change	13 (13)
Respiratory organ, thorax, and mediastinal disorders	11 (11)
Infections	12 (12)
Renal and urinary tract disorders	11 (11)
Vascular complications	9 (9)
Elevated transaminases	5 (5)
Other adverse events	6 (6)
Causes of LCPT discontinuation, *n* (%)	
Hair loss	1 (1)
Pruritus	1 (1)
Tremor	1 (1)
Alopecia areata, tremor, reduced finger sensitivity, restlessness, sleep disorders	1 (1)
Supratherapeutic tacrolimus levels	1 (1)
Death	2 (2)
Lost to follow-up	2 (2)

LCPT, Once-daily MeltDose^®^ Extended-Release Tacrolimus.

## Data Availability

The data generated in this study is available upon request from the corresponding author. All requests will be reviewed to ensure compliance with applicable security protocols and data protection regulations.

## Abbreviations

The following abbreviations are used in this manuscript:

ALT	Alanine Transaminase
AP	Alkaline Phosphatases
AST	Aspartate Transferase
BMI	Body Mass Index
CI	Confidence Interval
CNI	Calcineurin Inhibitor
ESC	Essen Compliance Score
GFR	Glomerular Filtration Rate
GGT	Gamma-glutamyl Transferase
IR-Tac	Immediate Release Tacrolimus
LCPT	MeltDose^®^ Extended-release Tacrolimus
LT	Liver Transplant
PLTDM	Post-liver Transplantation Diabetes Mellitus
PR-Tac	Prolonged-release tacrolimus
